# In-hospital complications after invasive strategy for the management of Non STEMI: women fare as well as men

**DOI:** 10.1186/1471-2261-10-31

**Published:** 2010-06-24

**Authors:** Caroline Berthillot, Dominique Stephan, Michel Chauvin, Gerald Roul

**Affiliations:** 1 Pôle d'Activité Médicochirurgicale Cardiovasculaire, Unité de Soins Intensifs Cardiologiques - Nouvel Hôpital Civil, Place de l'Hôpital, 67000 Strasbourg - France

## Abstract

**Background:**

To analyze the in-hospital complication rate in women suffering from non-ST elevation myocardial infarction treated with percutaneous coronary intervention (PCI) compared to men.

**Methods:**

The files of 479 consecutive patients (133 women and 346 men) suffering from a Non STEMI (Non ST-segment elevation myocardial infarction) between the January 1^st ^2006 and March 21^st ^2009 were retrospectively analyzed with special attention to every single complication occurring during hospital stay. Data were analyzed using nonparametric tests and are reported as median unless otherwise specified. A p value < .05 was considered significant.

**Results:**

As compared to men, women were significantly older (75.8 *vs*. 65.2 years; p < .005). All cardiovascular risk factors but tobacco and hypertension were similar between the groups: men were noticeably more often smoker (p < .0001) and women more hypertensive (p < .005). No difference was noticed for pre-hospital cardiovascular drug treatment. However women were slightly more severe at entry (more Killip class IV; p = .0023; higher GRACE score for in-hospital death - p = .008 and CRUSADE score for bleeding - p < .0001). All the patients underwent PCI of the infarct-related artery after 24 or 48 hrs post admission without sex-related difference either for timing of PCI or primary success rate. During hospitalization, 130 complications were recorded. Though the event rate was slightly higher in women (30% *vs*. 26% - p = NS), no single event was significantly gender related. The logistic regression identified age and CRP concentration as the only predictive variables in the whole group. After splitting for genders, these parameters were still predictive of events in men. In women however, CRP was the only one with a borderline p value.

**Conclusions:**

Our study does not support any gender difference for in-hospital adverse events in patients treated invasively for an acute coronary syndrome without ST-segment elevation and elevated troponin.

## Background

Percutaneous Coronary Intervention (PCI) is the preferred technique for the treatment of acute coronary syndrome with or without ST-segment elevation according to the Guidelines [1]. The available data raise concerns about sex differences in outcome after invasive treatment for acute coronary syndromes (ACS). However every day, PCI is offered to everybody suffering from ACS regardless of its gender. In the study reported by Bell *et al. *in 1993 [2] from a large cohort of 2955 men and 1106 women, a greater in-hospital mortality was recorded in women and was related, at least partly, to the severity of the underlying disease rather that gender alone. On the other hand, no gender related differences in outcome was found by Mehilli *et al *[3] after PCI in women and men who underwent stent placement for stable angina. Malenka and co-workers [4] analyzed the impact of PCI techniques improvement on sex difference in outcome and found no sex specificity in a sample of 33666 patients of both sex. These authors [4] related their results upon the improvement in PCI procedures overtime. More recently, Berger *et al *[5] analyzed data issued from 11 studies merged in one single database. They reported on 30-day mortality following ACS and found no gender related difference after adjustment for baseline variables. More recently, the sex difference in outcome following PCI have been reassessed in a report from Duvernoy et al [6] about a large population of 24,725 patients (31.8% were women) from 17 hospitals in Michigan between January 2002 and December 2003. PCI was offered in every case for various clinical presentations. They concluded that differences in mortality rates between men and women no longer exist after PCI. They also suggested that technological advancements have not completely offset the relationship between gender and adverse outcomes after PCI. All these studies included different clinical presentations of coronary artery disease with a mixed of ST- segment elevation myocardial infarction, Non ST-segment elevation myocardial infarction (NSTEMI), unstable and stable angina, a heterogeneity which could minimized a difference to some extent. PCI data about sex related differences are limited in the area of NSTE-ACS (non ST elevation-acute coronary syndrome), a growing subset of coronary artery disease with an annual incidence of hospital admission of 3 per 1000 inhabitants [1]. Therefore, we aimed to compare the in-hospital outcome between men and women with NSTEMI only and treated with PCI.

## Methods

### Patients and data collection

The files of all consecutive patients admitted to our university hospital with the diagnosis of NSTEMI (code 410.7 from ICD-9) between January 1^st ^2006 and March 21^st ^2009 was retrospectively analyzed. The diagnosis of NSTEMI was made according to the classical criteria enacted by the 2007 Guidelines of the European Society of Cardiology [1] taking into account clinical data, ECG changes and raise of cardiac troponin I plasma level.

We collected from the medical files all administrative data: last name, first name, gender, date of birth, time to angioplasty, length of stay; the past medical history (cardiovascular and non cardiovascular); cardiovascular risk factors: body mass index, diabetes mellitus, hypertension, dyslipidemia, smoking habits, family history of premature cardiovascular disease and anxiety. The clinical characteristics (blood pressure, pulse pressure, heart rate, Killip class) were also noted as well as the biological data (hemoglobin, hematocrit, platelets count, hemostasis, higher plasma troponin as a surrogate of infarct size, B-type natriuretic peptide (BNP) or N Terminal pro-BNP, plasma glucose at entry, Glycated hemoglobin, CRP (C reactive protein), plasma creatinine from which glomerular filtration rate was derived from the Cockcroft formula [7]; ECG parameters (sinus rhythm or not and ST-T changes); left ventricular ejection fraction derived from 2-dimensional echocardiography [8] and the PCI procedure reports for the extension of the coronary artery disease, number of stents and all other procedure related specificities - *e.g*. number of coronary arteries treated, amount of contrast media administered. We also recorded the number of succeeded or failed procedures and all reported complications (cardiac, hemorrhagic, renal, embolic, infectious events) as well as death during the hospital stay. The appropriate GRACE likelihood for in-hospital death and complications [9,10] and the CRUSADE bleeding score [11] were also entered. The ethical committee of our institution approved the study design and data collection.

### Statistical analysis

Data are presented as median and inter quartile range, unless otherwise specified. The normality of the distribution of all continuous variables was checked using the D'Agostino and Pearson omnibus normality test. In case of non normal distribution, continuous data were compared using the Mann and Whitney test in case of unpaired values and with the Wilcoxon signed rank test in case of paired data. The time course of some continuous parameters was also assessed using a nonparametric ANOVA for repeated measures: the Friedman test with localization of differences by Dunn's multiple comparison test. Proportions were compared using the Chi-Square test with Fischer exact test if necessary after. The *p *value was considered significant if less than 0.05. The statistical power was calculated using a 'not significant' chi-square test comparing two proportions. All statistical analyses but one were performed using the PRISM package (Prism 4.0c and Statemate for Macintosh; GraphPad software Inc., USA). Logistic regression was performed with the Statview for Windows software (SAS Institute 1992-98; Version 5.0).

## Results

### Population

The study population consisted in 479 patients, with a predominance of men (72%). Patients' characteristics are summarized in Table 1. Women represented 28% of our sample. They were significantly older (p < 0.005). Among the cardiovascular risk factors, one can notice that women were less often smokers (p < 0.0001) and had more hypertension (p < 0.005). Though diabetes mellitus was more often reported in men with a borderline *p *value, the glycated hemoglobin level was higher in women (p < 0.03) likely related to a poorer control of their diabetes. All other reported parameter showed no gender related difference (Table 1).

**Table 1:** Demographic data

	Men (N = 346)	Women (N = 133)	p Value
Age (yrs)	65.2 (56.3-75.6)	75.8 (66-80.5)	0.0034
BMI (kg/m^2^)	27.1 (17-31)	27.1 (17-30)	NS
Dyslipemia (%)	36.1	43.6	NS
Total cholesterol (mg/dl)	1.71 (1.39-2.02)	1.65 (1.47-2.14)	NS
LDL cholesterol (mg/dl)	1.04 (0.77-1.31)	0.97 (0.7-1.36)	NS
HDL cholesterol (mg/dl)	0.4 (0.3-0.43)	0.4 (0.3-.53)	NS
Diabetes (%)	34.1	21.0	0.0574
Smoker (%) [current]	70.8 [34.1]	33.1 [18]	< .0001
Hypertension	64.7	78	0.0043
Family history (%)	17.1	10.8	NS
Anxiety (%)	2.31	4.60	NS

Abbreviations: BMI: body mass index. Current in brackets stands for active smokers. Family history stands for a family history of premature cardiovascular disease. yrs: years.

Considering the past medical history (Table 2), no difference was found for the rate of pre-existing cardiovascular disease of any etiology, atrial fibrillation, peripheral arterial disease and stroke or pacemaker holders. Non-cardiovascular past history was also similar in both genders except for chronic obstructive pulmonary disease, which was significantly more frequent in men (p < 0.04). Likewise, the patients' pre-hospital drug treatment was similar whatever the therapeutic class considered: angiotensin converting enzyme inhibitors; angiotensin receptor blockers; beta-blockers; antiarrhythmic agents; calcium channel blockers; lipids lowering agents (statins and fibrates); antithrombotic agents either antiplatelets or anticoagulants; diuretics; oral antidiabetic agents also accounting for metformine; insulin; nitrates. For angiotensin system antagonists, beta-blockers and statins, not only the rate of prescription but also the optimal doses were similar between genders. The rate of prescription of antidepressants was however significantly different, women being more likely to receive this class of drugs than men (p < 0.03).

**Table 2:** Past history of cardiovascular diseases

	Men(N = 346)	Women(N = 133)	p Value
Non-ischemic heart disease (%)	2.6	11.3	NS
Ischemic heart disease (%)	37	30.8	NS
Left main	1.2	3.8	NS
LAD	27.5	27.1	NS
Circumflex or 1^st ^Marginal	20.8	21.8	NS
Right coronary artery	25.7	19.6	NS
Coronary Artery Bypass Grafting (%)	9.3	6.8	NS
LAD	8.4	4.5	NS
Circumflex or 1^st ^Marginal	1.7	0.8	NS
Right coronary artery	3.8	2.3	NS
Atrial Fibrillation (%)	7.8	8.3	NS
Peripheral Arterial Disease (%)			
Lower Limb	17.1	17.3	NS
Carotid	12.7	10.5	NS
Venous thrombo-embolic diseases (%)	3.5	11.3	0.0017
Pace Maker (%)	4.1	3.8	NS
Stroke (%)	8.4	9.8	NS

Abbreviations: LAD: left descending coronary artery.

### Clinical characteristics

The clinical characteristics on admission are described in Table 3. As compared to men, women had a higher pulse pressure (68 *vs*. 60 mm Hg - p < 0.05). Pulse pressure was slightly but significantly related to age in men (rho = 0.15; p = 0.008). In women, this wasn't the case (rho = 0.14; p = 0.1 - NS). Moreover, women had also more severe heart failure. Indeed, 6.5% of women were in Killip class IV and only 0.9% of men (p < 0.003). This difference could be, at least partly, related to the higher rate of right heart failure in women (24% of our female population; p < 0.02). This trend to a less favorable clinical profile on admission was also suggested by the probability of in-hospital death derived from the appropriate GRACE probability (p < 0.01). The likelihood of in-hospital complications according to the specific GRACE probability was borderline (p = 0.068). The CRUSADE bleeding likelihood was clearly higher in women (11%; IQR: 8 - 15 *vs*. 6%; IQR: 4 - 10 in men; p < 0.02). In spite of the more compromised hemodynamic profile in women, no difference could be noted for mean arterial pressure or heart rate between the groups (Table 3).

**Table 3:** Clinical data at entry

	Men (N = 346)	Women (N = 133)	p Value
**Blood pressure **(mmHg)			
MAP	107 (97-119)	106 (95-120)	NS
PP	60 (50-74)	68 (50.5-80)	0.0477
**HR **(bpm)	75 (64-85)	76 (64-85)	NS
**Killip class**			
I (%)	65.5	61.5	NS
II (%)	25.07	22.3	NS
III (%)	8.6	10	NS
IV (%)	0.9	6.15	0.0023
**Right heart failure **(%)	13.7	23.8	0.0119
**GRACE likelihood (In-hospital)**			
Death (%)	2 (1-6)	3 (1-8)	0.0080
Complications (%)	14 (9-20.0)	16 (10-22.5)	0.0678
**CRUSADE likelihood**	6 (4-10)	11 (8-15)	< 0.02

Abbreviations: bpm: beats per minute. HR: heart rate. MAP: mean arterial pressure. PP: pulse pressure.

ECG changes in anterior leads only were similar in both groups. ECG changes in all lateral leads were more frequent in women (6.8% *vs*. 2.3%; p < 0.03) whereas ECG changes solely in inferior leads were more common in men (11.8% *vs*. 5.2%). Most of the patients had combined ECG changes: 53% of women and 47% of men - p = NS. One third of the women population and 33.5% of men had no ECG changes at entry without any statistical difference. These patients remained in the study on the basis of clinical presentation and elevation of plasma troponin I. One atrioventricular block was recorded at entry in each group. No difference was noted for the presence of atrial fibrillation on admission (6.1% in men and 7.5% in women; p = NS). Apart from isolated lateral and inferior leads concerning a few patients, no difference could be noted between the groups.

### Biological characteristics

**Figure 1:**
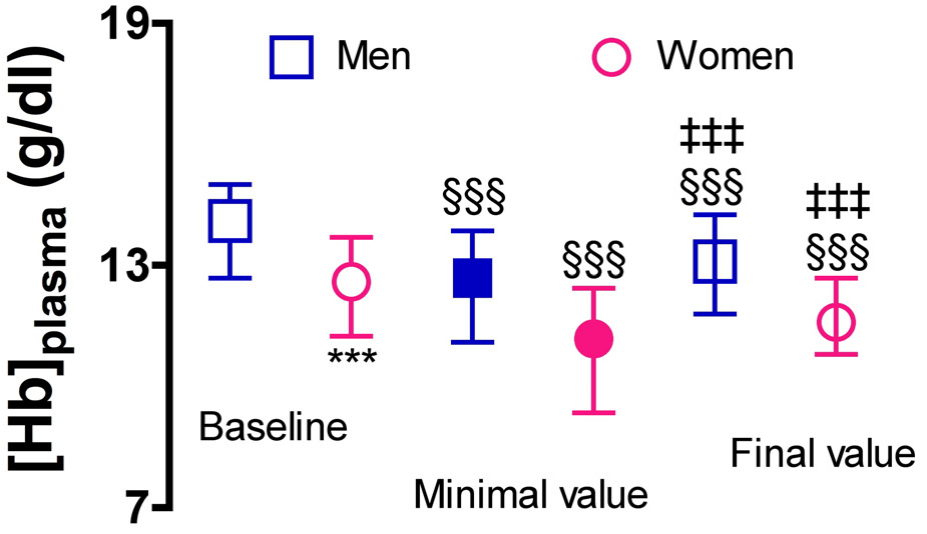
**Time course of hemoglobin during hospital stay**. Compared to baseline, both group as the same course as suggested by a 2-way ANOVA showing no significant group interaction.  §§§: p < 0.0001 compared to baseline. ‡‡‡: p < 0.0001 compared to minimal value.  ***: p < 0.0001 between women and men.

The biological characteristics at hospital entry are displayed in Table 4. Many classical parameters as well as Troponin I and BNP plasma level were similar between genders. Plasma glucose was significantly higher in women (p < 0.05). Hence, women also had more inflammation as suggested by both CRP (p < 0.05) and plasma fibrinogen (p < 0.002). In women we noted lower levels of hemoglobin (p < 0.0001) and of hematocrit (p < 0.0001) however with the same time course during hospitalization in both groups (Figure 1). Baseline renal function was reduced in women (p < 0.0001).

**Table 4:** Biological data at entry

	Men (N = 346)	Women (N = 133)	p Value
Platelets count (n*10^3^/l)	229 (192-278)	252 (201-295)	NS
Hemoglobin (g/dl)	14.0 (12.7-15.0)	12.6 (11.3-13.7)	0.0001
Hematocrit (%)	40.8 (37.3-43.3)	37.0 (33.6-40.2)	0.0001
APTT (s)	34 (32-39)	34 (31-39)	NS
BNP (ng/l)	104 (46-307)	159 (67-386)	NS
eGFR (ml/min)	82 (54-110)	57 (42-81)	< 0.0001
Troponin I (μg/l)	1.66 (0.46-6.72)	1.62 (0.47-6.8)	NS
Plasma glucose (g/l)	1.24 (1.03-1.69)	1.33 (1.10-2.03)	< 0.05
Glycated Hemoglobin (%)	6.1 (5.7-7.1)	6.6 (5.8-7.7)	0.0204
CRP (mg/l)	4.3 (4.0-12.5)	26.7 (7.5-71.0)	< 0.05
Fibrinogen (g/l)	3.8 (3.2-4.7)	4.2 (3.2-5.2)	< 0.002

Abbreviations: APPT: activated partial thromboplastin time. BNP: B-natriuretic peptide. CRP: C Reactive Protein. eGFR: estimated glomerular filtration rate.

### PCI procedure

All the patients underwent a coronary angiogram with PCI in a similar median time (Table 5). The contrast media used was ioxaglic acid in the vast majority of patients. The remaining was given either iodixanol or iomeprol. No difference existed between genders for iodixanol and Iomeprol doses. Men received substantially more ioxaglic acid (p < 0.02). This apparent excess in contrast volume did not translate into renal disorder as reflected by plasma creatinine and estimated glomerular filtration rate during hospitalization. Glomerular filtration rate at the end of hospitalization was 76 ml/min (IQR: 52 - 103) in men and 54 ml/min (IQR: 40 - 74) in women, not different from baseline values within groups.

**Table 5:** Contrast use, CAD extent and PCI procedures

	Men (N = 346)	Women (N = 133)	p Value
**Contrast agents**			
Ioxaglic acid (ml)	250 (200-340)	211 (180-260)	< 0.02
Iodixanol (ml)	220 (171-300)	200 (160-300)	NS
Iomeprol (ml)	215 (134-334)	226 (219-350)	NS
**CAD extent**			
1-vessel disease (%)	29	34	NS
2-vessel diseases (%)	36	34	
3-vessel diseases (%)	35	32	
**PCI procedure**			
Time interval to PCI (days)	1 (1-4)	2 (1-4)	NS
BMS (%)	51	61	0.052
DES (%)	40	32	NS
Balloon angioplasty (%)	9	14	NS
Thrombus aspiration (%)	1	0	NS
Use of closure devices (%)	83	81.5	NS
Failure (%)	5	8	NS
GPIIb/IIIa antagonists (%)	22	20	NS

Abbreviations: BMS: bare-metal stent. CAD: coronary artery disease. DES: drug eluting stent. GPIIb-IIIa antagonists: Glycoprotein IIb-IIIa antagonists. PCI: percutaneous coronary intervention.

The extent of coronary artery disease was similar between genders. Though there was no sex related difference for time to PCI and type of procedure, a tendency to more bare metal stent placement in women was noted (Table 5). Glycoprotein IIb-IIIa antagonists were used in 22% of men and 20% of women without any difference. Closure devices were equally used between the groups: 83% of men and 81.5% of women (p = 0.69) (Table 4).

### Outcomes

Left ventricular ejection fraction derived from 2-dimensional echocardiography after PCI was near normal and similar in both groups: 56%; IQR: 47 - 62 in women *vs*. 55%; IQR: 45 - 60 in men; p = NS). Length of stay was longer in women (median = 10 days; IQR: 5.5 - 17 *vs*. 7.5 days; IQR: 5 - 11; p < 0.01).

One hundred and thirty in-hospital complications were recorded: 90 in men and 40 in women. Thus 30% of women had experienced at least one complication as compared to 26% of men (p = NS).

The procedure was unsuccessful in 19 men and 11 women, but the difference was not significant (p = NS). In-hospital mortality was only 1.9% of the study population: 9 patients died (6 men and 3 women; p = NS). Among men, 1 died from a complete atrioventricular block, 2 from septic shock, 1 from cardiogenic shock and 2 from stent thrombosis. Among women, 1 died from severe congestive heart failure, 1 from septic shock and the last one from per procedure cardiac arrest. The septic shocks were all related to *Staphylococcus Aureus*. Nineteen patients presented severe hypotension requiring catecholamine support and 16 of them required transfer to the coronary care unit afterward (10 men *vs*. 9 women; p = NS). Two among these 19 collapses were a consequence of cardiac tamponade. All the others were related to severe congestive heart failure. The power of the study to detect a significant change of 0.04 between the groups is at least 80%.

Sixty-nine patients had a hemorrhagic event: 45 men and 24 women, p = NS. Most of the hemorrhagic events were confined to the femoral puncture point without gender difference: 17 women *vs*. 30 in men, p = NS. Other bleedings, including from gastrointestinal tract, were limited to a few patients (7 women *vs*. 15 men; p = NS). As a whole, hemoglobin loss was moderate: median value was -1.4 mg/dl; IQR = -2.2, -0.7 in women and -1.2 mg/dl; IQR = -2.1, -0.5 in men; p = NS). Only 39 patients (8% of the study population) were given blood transfusion without any sex related difference. As previously, the power of the study to detect a significant change of 0.10 between the groups for bleeding is at least 80%.

We also recorded some other complications figured in Table 6. These events were observed in 29% of women and 28% of men. Once more, no difference could be found between genders.

**Table 6:** Complications

	Men (N = 346)	Women (N = 133)	p Value
Death (%)	1.7	2.3	NS
Intensive care unit transfer (%)	2.3	6	0.0832
Counter pulsation intra aortic balloon (%)	1.4	1.5	NS
Collapse (%)	2.9	6.8	0.0662
Acute pulmonary edema (%)	1.5	0.8	NS
Bleeding (%)	13	18	NS
Recurrent ischemia (%)	2.6	1.5	NS
Stent thrombosis (%)	1.5	1.5	NS
Arrhythmia/conduction disturbances (%)	3.8	3	NS
Stroke (%)	0.3	0.8	NS
Infection (%)	6.1	7.6	NS
Allergic reaction (%)	1.2	2.3	NS

A logistic regression analysis taking into account sex, age, CRP, glomerular filtration rate, glycated hemoglobin, number of coronary vessel disease, pulse pressure, GRACE scores and CRUSADE score showed that the only independent predictive parameter was CRP (Chi-2 = 5.873; p = 0.0154). Some of these data were obviously correlated with age and sex (GRACE scores, CRUSADE score and Cockcroft formula). However, we wanted to include them in a first step in order to stress the strength of their relationship with the response variable and choose the best one. Afterward, the parameters remaining in the study were age, CRP, glycated hemoglobin, number of vessel disease, pulse pressure and sex. After removing from the model all variables but age, CRP and sex, because of significance level, we were left with 2 predictive variables: patients age: (Chi-2 = 13.141; p = 0.0003) and CRP (Chi-2 = 8.989; p = 0.0027) with a poor deviance goodness of fit as derived from its p-value (Chi-square = 499.044; p = 0.0212). After Splitting the result according to genders, this holds true for men: Chi-2 for age was 12.968 with a p value = 0.0003 and Chi-2 for CRP was 5.917 and p = 0.015. The deviance became not significant, suitable with a satisfactory Goodness of fit (deviance = 344.951; p = NS). In women, age was not significant anymore and CRP was of borderline significance (Chi-2: 3.562 and p < 0.06; deviance = 152.62; p = NS). However, keeping pulse pressure in the final model resulted in an increased weight for CRP in women: Chi-2 = 4.536 and p < 0.04 without noticeable change in men.

## Discussion

Our data showed no difference in outcome between men and women treated invasively for NSTEMI. The only parameters that matter are age and inflammation status, a possible reflection of a more advanced disease state. The problem of a worse outcome in women undergoing PCI has been underscored for a long time. In 1993, Bell and coworkers [2] reported a higher in-hospital and periprocedural mortality in women in a large sample of patients (n = 4071) after emergency PCI for STEMI, unstable angina or elective procedure for stable angina. In this study, genders were similar only for prior myocardial infarction and number of coronary vessel disease. All other variables were significantly different. They failed to find any strong relationship between female gender and outcome and came to the conclusion that their results could be more likely related to the severity of the underlying disease. Though their sample size was large, the population was heterogeneous and could have undermined their findings. Our data, obtained from a series of homogeneous patients are online with their conclusions. There is no obvious sex related effect *per se *explaining any difference in outcome. More recently, Mehilli and others [3] reported about a series of 1001 women and 3263 men treated with coronary stenting for symptomatic coronary artery disease in the nineties on an elective basis. They did not mention the in-hospital outcome but reported a higher 30-day event rate in female gender, though this difference had disappeared at 1 year. They acknowledged that some heterogeneousness in baseline characteristics could have explained their findings. Our data emphasize this aspect. When one can reduce the discrepancies between genders regarding most of the baseline characteristics, the outcome seems identical. The data reported by Malenka et al [4] referred to 33,666 patients having undergone PCI between 1994 and 1999. They had the basic concept that women were at higher risk for adverse outcomes after PCI. Though their female patients were older, had more diabetes, chronic obstructive pulmonary disease, renal failure, peripheral artery disease and heart failure, they found no sex related difference for post PCI coronary bypass grafting or myocardial infarction rate. They stated that the improvement in PCI techniques could explain their results. Even in a more contemporary era, PCI techniques are still improving and we cannot rule out such a possibility, as we could not analyze this aspect. However, the progress made during the study period that could account for our results are unlikely. There was just a trend to have more women implanted with bare-metal stents and the rate of drug-eluted stents was similar between genders. In no way the number of complications was influenced by the type of stent. The conclusion we made is also suggested by a recent study from Berger *et al *[5] who investigated the relationship between gender and 30-day mortality in acute coronary syndromes. The differences they found were largely explained by differences at presentation and severity of the coronary artery disease. Weintraub *et al. *[12] also reported a higher in-hospital mortality post PCI in women. However, in the multivariate analysis, the correlates of in-hospital death were by far the severity of the disease state whereas there was only a trend for female gender [12]. Welty *et al. *[13] found no difference between genders for procedural outcome of PCI and both sex had a similar success rate in their study. Schuhlen *et al. *[14] also found that female gender had no excess risk for major adverse cardiac events after intracoronary stenting. Our data confirm that women fared as well as men for success rate after PCI for NSTEMI. Indeed, failing procedures were low in both groups.

Even though we found no sex related difference for outcomes, the women we studied were ten years older and had clearly more hypertension and a trend to lower rate of diabetes mellitus. They were less likely smokers. These features are well established not only in coronary artery disease but also in heart failure [15,16] and reports dealing with gender related differences underlined these aspects [5]. Therefore, such characteristics don't change our conclusion. In spite of a lower rate of diabetes mellitus, women appeared to have significantly higher plasma glucose and glycated hemoglobin, likely linked to a poorer control of their diabetes. Plasma glucose at entry is predictive of in-hospital mortality as stated by several studies [17-21]. Hence, the cutoff value of impaired fasting glucose defining the risk for worse outcome has been specified by Vergès *et al. *[22] to be above 110 mg/dl. In our study, we only considered plasma glucose on admission. No correlation was evidenced with outcomes. Though statistically different, the gap was weak and could account for the reported result. Neither plasma glucose at entry nor glycated hemoglobin remained after the multivariate analysis. Our data are corroborated by the paper from Vivas *et al. *[23] and from Suleiman *et al. *[24] who found that admission plasma glucose had no predictive value unlike the first fasting plasma glucose that was not analyzed in our study.

There are several scores for risk prediction at hospital entry, each of which facilitates patient management with ACS - GRACE [9], TIMI [25], PURSUIT [26] -. All these scores have a good predictive value to identify high-risk subsets of patients who will benefit most from myocardial revascularization performed during initial hospitalization [27]. For this study we have chosen the in-hospital GRACE risk score which is widely used in the setting of NSTEMI and seems perhaps better than the equivalent TIMI score [28,29]. We noticed that women had a significantly higher GRACE risk score for death and a trend to a greater likelihood for in-hospital complications. However, we failed to confirm this difference in the multivariate analysis either for death or for overall complications. The in-hospital mortality was in agreement with other reports [24]. Such discrepancy has been stressed in the literature [24,30]. Indeed, the predictive power of the GRACE score is well established. Nevertheless, it can be suboptimal is some cases and might require some recalibration as emphasized recently by Elbarouni *et al. *[31]. We didn't find genders difference for hemorrhagic complications either. The recently reported CRUSADE score we used [11] has been validated in the NSTEMI setting. This score takes female gender into account as risk marker for bleeding and is online with various reported data. The higher CRUSADE likelihood we found in women could be related to the weight factor of female gender in CRUSADE. Another reason for discrepancy might be linked to the way we analyzed bleeds. In CRUSADE, bleeding definition is according to GUSTO whereas we included all bleeds. We cannot rule out the effect of the high use of closure device to explain the recorded data.

We recorded a worse renal function in women, the negative prognostic value of which has been re-emphasized recently [32]. These authors [32] concluded from the large database of the SYNERGY trial, that chronic kidney disease derived from the estimated glomerular filtration rate was predictive of 30-day mortality, myocardial infarction and bleeding. We agree with this claim when age is not entered in our logistic model. Once this was done, estimated glomerular filtration rate retained no more value.

In our patients, we analyzed the inflammation status as various reports underscored the interest of doing so [33-36]. Considering the role of inflammation in the initiation, progression and destabilization of atheroma, the reported result is not surprising. Our data on CRP and prediction of complications including death in NSTEMI patients are in harmony with other results summarized by Blake and Ridker [35] and others [37,38].

### Study Limitation

We studied a sample of NSTEMI according to the available guidelines. However, the sample size is small though comparable to others. Our study is from one site and included only 28% of female gender and was of retrospective nature. Though we do believe that female gender is not at higher risk for PCI in NSTEMI, more data are needed to draw final conclusion.

## Conclusions

In spite of a higher risk profile in female as stated by various well-established parameters, female patients treated with PCI for Non STEMI have the same in-hospital course outcome than men. The only predictive variables for adverse events were age and inflammation status. This latter could be the expression of the stage of the underlying pathological process at least to some extent.

## Competing interests

The authors declare that they have no competing interests.

## Authors' contributions

CB collected, analyzed the data and wrote the manuscript. GR designed the study, performed the statistical study, and contributed to the analysis of the data and the writing of the manuscript. DS and MC read the manuscript and contributed to its improvement. All authors have read and approved the final manuscript.

## Pre-publication history

The pre-publication history for this paper can be accessed here:

http://www.biomedcentral.com/1471-2261/10/31/prepub
